# Only cervical vertebrae C0-C2, not C3 are relevant for subgrouping migraine patients according to manual palpation and pain provocation: secondary analysis of a cohort study

**DOI:** 10.1186/s12891-022-05329-2

**Published:** 2022-04-22

**Authors:** Annika Schwarz, Kerstin Luedtke, Thomas Schöttker-Königer

**Affiliations:** 1 Faculty of Social Sciences, University of Applied Sciences Bremen, Am Brill 2-4, 28195 Bremen, Germany; 2 Department of Systems Neuroscience, University Medical Center Hamburg-Eppendorf, Hamburg, Germany; 3 Department of Physiotherapy, Pain and Exercise Research Luebeck (P.E.R.L), Institute of Health Sciences, Universität Zu Luebeck, Luebeck, Germany; 4 Faculty of Social Work and Health, University of Applied Sciences and Arts Hildesheim, Hildesheim, Germany

**Keywords:** Stratification, Migraine patients, Manual palpation, Cervical spine, Principal component analysis

## Abstract

**Background:**

Subgrouping of migraine patients according to the pain response to manual palpation of the upper cervical spine has been recently described. Based on the neuroanatomy and the convergence of spinal and trigeminal nerves in the trigeminocervical complex, the cervical segments C1 to C3 are potentially relevant. To date it has not been investigated whether palpation results of all upper cervical segments are based on one underlying construct which allows combining the results of several tests. Therefore, the aim of this secondary analysis of a cohort study was to determine whether results from all three segments form one construct.

**Methods:**

Seventy-one migraine patients with chronic or frequent episodic migraine diagnosed according to the international headache society classification version 3 were examined by one physiotherapist. Manual palpation using a posterior to anterior pressure was performed on the upper three cervical vertebrae unilaterally left and right. The results of the palpation according to the patients’ responses were combined using factor analysis. In addition, item response theory (IRT) was used to investigate the structure of the response pattern as well as item difficulty and discrimination.

**Findings:**

Factor analysis (principal component) showed that the palpation of C3 loads less onto the underlying construct than the palpation of C1 and C2. Considering a cut-off value > 1.0, the eigenvalues of all three segments do not represent one underlying construct. When excluding the results from C3, remaining items form one construct. The internal consistency of the pain response to palpation of C1 and C2 is acceptable with a Cronbach’s alpha of 0.69. IRT analysis showed that the rating scale model fits best to the pain response pattern. The discrimination value (1.24) was equal for all items. Item difficulty showed a clear hierarchical structure between the palpation of C1 and C2, indicating that people with a higher impairment are more likely to respond with referred pain during palpation of C2.

**Conclusion:**

Statistical analysis confirms that results from the palpation of the cervical segments C1 and C2 in migraine patients can be combined. IRT analysis confirmed the ordinal pattern of the pain response and showed the higher probability of a pain response during palpation of C2. The pain response to C3 palpation is not relevant for unidimensional IRT analysis.

**Trial Registration:**

German registry of clinical trials (DRKS00015995), Registered 20. December 2018, https://www.drks.de/drks_web/setLocale_EN.do

## Introduction and background

Migraine patients showed significantly more musculoskeletal findings in the cervical spine (Cx) compared to a control group without headaches [1]. The so-called trigeminocervical complex (TCC) has been established as a model that explains this relationship via a connection between the trigeminal system (trigeminal nerve) and the cervical system (greater occipital nerve—GON). The hypothesized model is based on a convergence of trigeminal and cervical afferents at brainstem level [2, 3]. The trigeminocervical convergence theory also potentially explains an influence of cervical structures on migraine symptoms.

### Upper cervical spine and migraine

The presence of additional neck pain is associated with a more severe clinical presentation in migraine patients and a delayed response to pharmacological therapy [4, 5]. Migraine patients with neck pain are more likely to show allodynia, decreased C1/C2 cervical range of motion, and poorer function of deep neck flexor muscles [4]. Furthermore, migraine patients frequently show symptomatic upper cervical joints [6] and increased neck muscle tension is associated with more severe attacks, shorter interictal periods, and a higher risk of continuous headache [7]. Limitations in upper cervical spine mobility, tested with the flexion-rotation test, showed a correlation with a higher frequency of headaches [8]. Patients with chronic migraine are significantly more limited and respond with a higher pain provocation during this test [8]. The importance of the upper cervical spine in migraine patients seems therefore evident.

Therefore, palpation of the upper cervical spine might be a suitable test for distinguishing migraine patients from healthy individuals and previous work has shown that migraine patients can be stratified into three groups based on pain provocation [9]:

1. no pain response
2. local pain provocation
3. pain radiating into the typical headache area

These results indicate that there are potentially different underlying pathophysiological phenomena within the migraine population, in which presumably the influence of the cervical spine on migraine symptoms is either particularly strong, possibly strong, or not existing. Analogous findings have also been described in previous studies [10]. To stratify patients in a previous study [9], they were allocated to the local pain group when at least one of the palpation points was painful and allocated to the group with referred pain to the head as soon as they reported referred pain to the head during palpation of at least one point.

However, all previous studies have focused on the vertebrae C0-C2 (i.e., occiput/C1 and C1/C2) of the upper cervical spine. Ignoring C3 (i.e., segment C2/C3), might provide misleading results. Anatomically, the nucleus trigeminus spreads as far caudally as C3. Based on the neuroanatomical relationship described above, all segments including C3 should be assessed. Structures that are capable to produce referred pain to the head are innervated by C1, C2 and C3 spinal nerves [11]. To determine whether it is justified to use the pain response to multiple palpation points collectively to form a subgroup according to the response, it must be verified that they form one underlying (latent) construct. Therefore, it must be investigated whether the test results of C3, C2 and C1 can be summarized for the stratification of migraine patients. The aim of this secondary analysis of a cohort study [12] was therefore to analyze whether palpation points at C1-3 form one underlying construct using item-response-theory (IRT).

## Methods

Methods in detail are described elsewhere [12]. In brief the methods were as follows:

### Participants

#### Inclusion criteria

Participants had to have chronic or frequent episodic migraine diagnosed by physicians at the Headache Outpatient Clinic of the University Medical Center Hamburg Eppendorf using the IHS (International Headache Society) third edition criteria [13]. In the main study, patients were included if their treating physician decided to offer a GON block for the prevention of migraine. They had to be at least 18 years old and have kept a headache diary for at least the previous four weeks.

#### Exclusion criteria

Patients who had a diagnosed pathology of the cervical spine with existing symptoms or a history of trauma (e.g., whiplash) to the cervical spine were excluded. Furthermore, patients during pregnancy or lactation were excluded. Patients were not allowed to have a medically relevant internal, psychiatric, or neurological disease that could have affected pain perception. Patients with alcohol and/or substance abuse in their medical history were also excluded.

### Study procedures

The criteria of the Declaration of Helsinki [14] and the criteria of good clinical practice [15] were followed. The study was registered in the German registry of clinical trials (DRKS00015995) before study initiation. Patients were recruited consecutive from October 2018 to December 2019. Written informed consent was obtained on the day of the examination. All examinations were performed by one examiner. During the examination, a standardized procedure was ensured. The investigator was a physiotherapist and manual therapist (OMPT—orthopedic manual physiotherapy) with 15 years of clinical experience.

### Manual joint testing—subgrouping

**Fig. 1:**
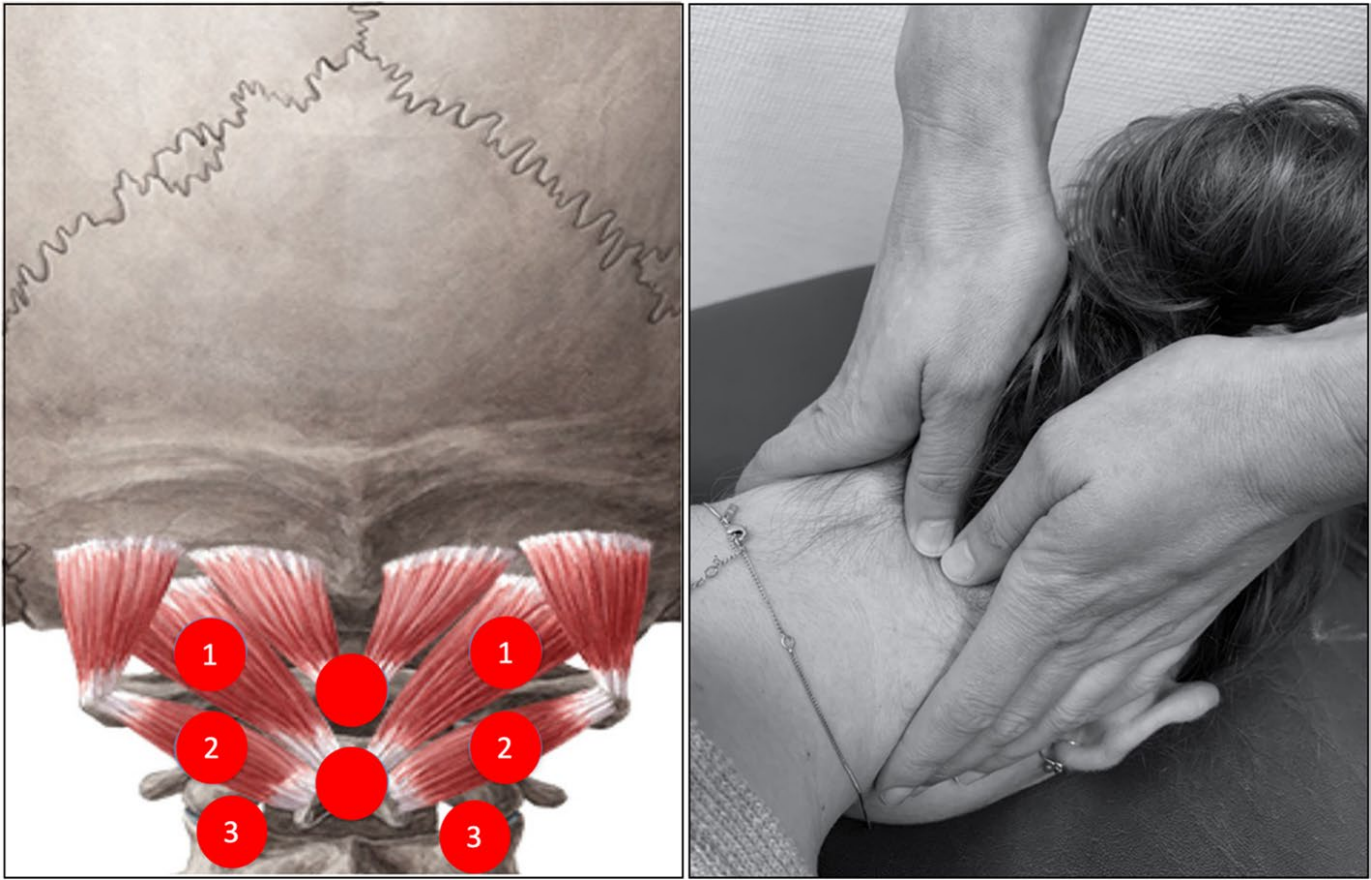
Adapted palpation points of the upper cervical spine from Luedtke and May, 2017. Left Palpation points of the cervical segments C0—C3: 1: Atlanto-occipital joint = pressure on transvers process of C1; 2: Atlanto-axial joint = pressure on transvers process of C2; 3: Joint C2/C3 = pressure on transvers process of C3 [9]; right Execution of the technique (own image)

Manual joint testing was performed for subgroup stratification into "no pain," "local pain," and "referred pain to the head" groups, using a protocol based on the study by Luedtke and May [9]. Gentle pressure was applied with both thumbs to the points marked in Fig. 1 to successively produce a ventral glide. The protocol was adapted to extend the examination to the C2/3 segment. Only unilateral techniques were used because these were found to be more informative in a previous trial [9]. Thus, palpation point "3" with pressure on the transvers process of C3 was added to include segment C2/C3 (Fig. 1). The patient was asked to report whether the pressure applied was painful and, if so, whether it radiated towards the head. The pressure was then held for approximately five seconds and again patients were asked for pain provocation. If the pressure was too uncomfortable for the patient, the test was terminated. This test procedure corresponds to the standard manual therapy examination of the upper cervical spine according to Maitland [16]. The tests with sustained pressure were described by Watson and Drummond for headache patients [10]. The best possible sensitivity and specificity are obtained in the combination of palpation and held pressure with the patient’s response regarding pain provocation [9].

### Data analysis

For the stratification, the pain responses to all palpation points were combined. However, if several measurable variables are combined into one value, it is assumed that they measure the same hypothetical or latent construct [17]. In this case, the measured latent construct can be described as “pain response to upper cervical palpation”. Thereby, it is assumed that segments with a painful response to palpation reflect the same degree of upper cervical dysfunction and that dysfunction is a continuum ranging from "no dysfunction" to "high dysfunction"—the stronger the dysfunction, the stronger the pain response.

Firstly, the hypothesis of unidimensionality of the palpation points of the segments C1 to C3 was tested. Therefore, a factor analysis (principal component analysis (PCA)) of all six palpation responses was performed based on a Spearman's rank correlation [18, 19]. An eigenvalue of > 1.0 was set as a cut-off value, as recommended by Field [18], to determine whether or not a component should be included.

Then IRT modelling was used to assess the psychometric properties of the underlying construct and related items. Because of the ordinal response options of the single items (palpation responses) polytomous item response theory (IRT) models were used [20]. First, the best fitting model was selected using Akaike’s information criteria (AIC), the Bayesian information criterion (BIC) and the Likelihood ratio test (LRT) [20]. Psychometric properties, including internal consistency and item characteristics were calculated. Internal consistency was estimated using Cronbach´s alpha. Acceptable internal consistency is reached if alpha is > 0.7 [17]. After fitting the IRT model, item characteristics, including item hierarchy, item difficulty and item discrimination, were calculated. The item hierarchy was observed to investigate whether the items were ordered in a logical manner, allowing the assessment of construct validity. Item difficulty parameters describe the points where the probability of choosing one response option versus the next is 50% (midpoint probability). Item difficulty parameters can be used to investigate item hierarchy, they are displayed in the boundary characteristic curve [20]. Category ordering was assessed to determine how the sample used the rating scale. Each item has three response categories (0 = no pain, 1 = local pain, 2 = referred pain) and thus 2-steps calibration thresholds at which the likelihood of endorsing one response option is equal to that endorsing the next category. Item ordering is visualized in the category characteristic curve. The item discrimination parameter describes the slope of the item characteristic curve. Higher values are indicating better item discrimination. Values > 1 are desirable [20]. Because of the small sample size, bootstrapping with 500 replications was used to estimate the IRT rating scale model [21]. Statistical analysis was performed with the IBM Statistical Package for Social Science (SPSS Version 25, Armonk, NY, USA) and Stata/IC Version 16.1 (StataCorp, Texas, USA). To avoid potential bias data collection and data analysis were performed by different persons.

## Results

### Participants – demographic data 

Seventy-one patients with chronic migraine (*n* = 56) and frequent episodic migraine (*n* = 15) met the inclusion criteria. Overall, 89% of participants were female, 65% reported headache during examination, and 17% were examined interictally (Demographic data is shown in Table 1).

**Table 1:** Demographic Data

Variables	Mean / number	SD/ percentage
diagnosis		
CM	56	79%
EM	15	21%
interictal	12	17%
Age (years)	44.01	13.5
sex		
female	63	89%
male	8	11%
BMI	24.9	4.9

Mean and standard deviation (SD) or number and percentage distribution of diagnosis and sociodemographic data. *CM* chronic migraine, *EM* episodic migraine, *BMI* Body mass index

### Determination of the palpation points to be considered—principal component analysis and component matrix

The principal component calculation with the items of all six palpation points from C1 to C3 showed that in the component matrix the palpation points of C3 loaded less on the principal component than the four palpation points of C1 and C2 (Table 2A). Looking at the associated eigenvalues, component two also had an eigenvalue > 1.0 (Table 3) and was thus above the cut-off value for exclusion [18]. It cannot be clearly determined with only six palpation points whether one or two components are represented. Thus, the points did not clearly measure only one construct. For subgroup assignment, however, it is necessary to reduce to one construct.

**Table 2:** Component matrix with six and four palpation points

Component matrix A			Component matrix B	
**6 Palpation Points**	**Component 1**	**Component 2**	**4 Palpation Points**	**Component 1**
Palpation C1 R	0.769	-	Palpation C1 R	0.829
Palpation C2 L	0.704	-	Palpation C2 L	0.750
Palpation C2 R	0.655	0.504	Palpation C1 L	0.701
Palpation C3 L	**0.516**	-	Palpation C2 R	0.584
Palpation C3 R	**0.576**	0.611		
Palpation C1 L	0.584	-0.590		

Component matrix A: loading of the six palpation points C1-3 unilaterally left (L) and right (R) on components 1 and 2, the items with the lowest loading are marked in bold; component matrix B: loading of the four palpation points C1-2 unilaterally left and right on component 1

**Table 3:** Principal component analysis and eigenvalues

C1 – C3				C1 – C2			
**Component**	**Initial Eigenvalues**			**Component**	**Initial Eigenvalues**		
	Eigenvalue	% of Varianz	Cummulated %		Eigenvalue	% of Varianz	Cummulated %
1	2.46	40.9	40.9	1	2.08	52.1	52.1
2	1.15	19.2	60.1	2	0.90	22.7	74.8
3	0.92	15.3	75.5	3	0.60	15.1	89.9
4	0.65	10.8	86.2	4	0.41	10.1	100
5	0.52	8.6	94.9				
6	0.30	5.2	100				
**Cronbach´s Alpha**	**0.71**			**Cronbach´s Alpha**	**0.69**		

The table presents the principal component analysis of the results of manual palpation of segments C1—C3 (left) and segments C1—C2 (right) and the corresponding Cronbach's alpha value. (C1/C2/C3: first/second/third cervical spine segment)

After removing the items that loaded less on the first component (C3 left and right, Table 2A), i.e., considering only the palpation points of C1 and C2, the principal component analysis indicated that only component one had an eigenvalue > 1.0 and was thus clearly different from the other components, whose eigenvalues were < 1.0 (Table 3). Accordingly, it can be assumed that one single construct was measured by the four items. The associated component matrix showed that all four items (palpation points of C1 and C2) loaded strongly on this one component (Table 2B). Consequently, the items measured the same underlying latent construct.

Thus, in the principal component analysis, it was shown that segments C1 and C2 measured the same latent construct and therefore the pain response of the four palpation points can be combined for group assignment. C3 on the other hand should not be considered for group assignment (Table 3). According to Field [18], Cronbach's alpha indicates an internal consistency for both construct measures that can be classified as acceptable (Table 3).

### Psychometric properties using IRT

The rating scale model (RSM) described by Andrich (1978) showed the best model-data fit statistics (lowest AIC and BIC values, not significant *p* value (*p* = 0.4321) when compared to the partial credit model) [22]. The RSM is a parsimonious IRT model where each item shares the same discrimination parameter, and the same responses have the same meaning across all items [20].

**Fig. 2:**
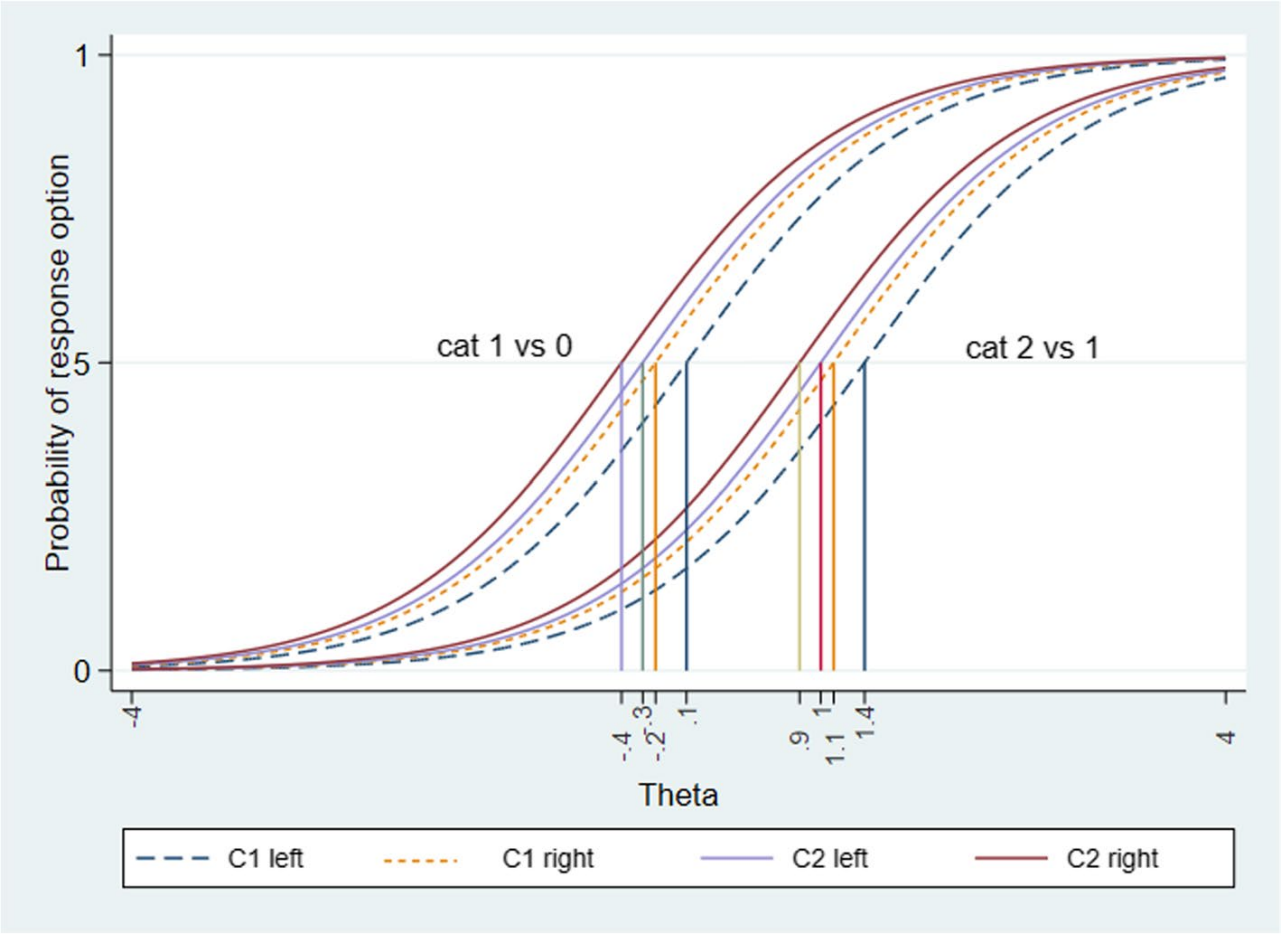
Boundary characteristic curve: Participants with a higher degree of dysfunction are placed towards the right side of the x-axis, Values given on the x-axis represents the difficulty parameters of choosing one category versus the next

After fitting the RSM, item hierarchy was investigated using the boundary characteristic curves (BCC) (see Fig. 2). The BCC displays the midpoint probabilities for choosing one response option versus the next one. The values given on the x-axis in Fig. 2 represent the difficulty parameters. The segment C2 had lower difficulty parameters than C1. This reflects that migraine patients of the current sample with a lower degree of cervical dysfunction (left side of the graph) were more likely to show a pain response to palpation on C2. Whereas only participants with a higher degree of dysfunction had a higher possibility to show a pain response (local pain or referred pain) on palpation of C1. Figure 2 also displays that the curves of all four items are parallel in their central part. That is because all four items share the same item discrimination parameter (discrimination value 1.24 95%CI:0.8–1.67) which is within the desirable level.

**Fig. 3:**
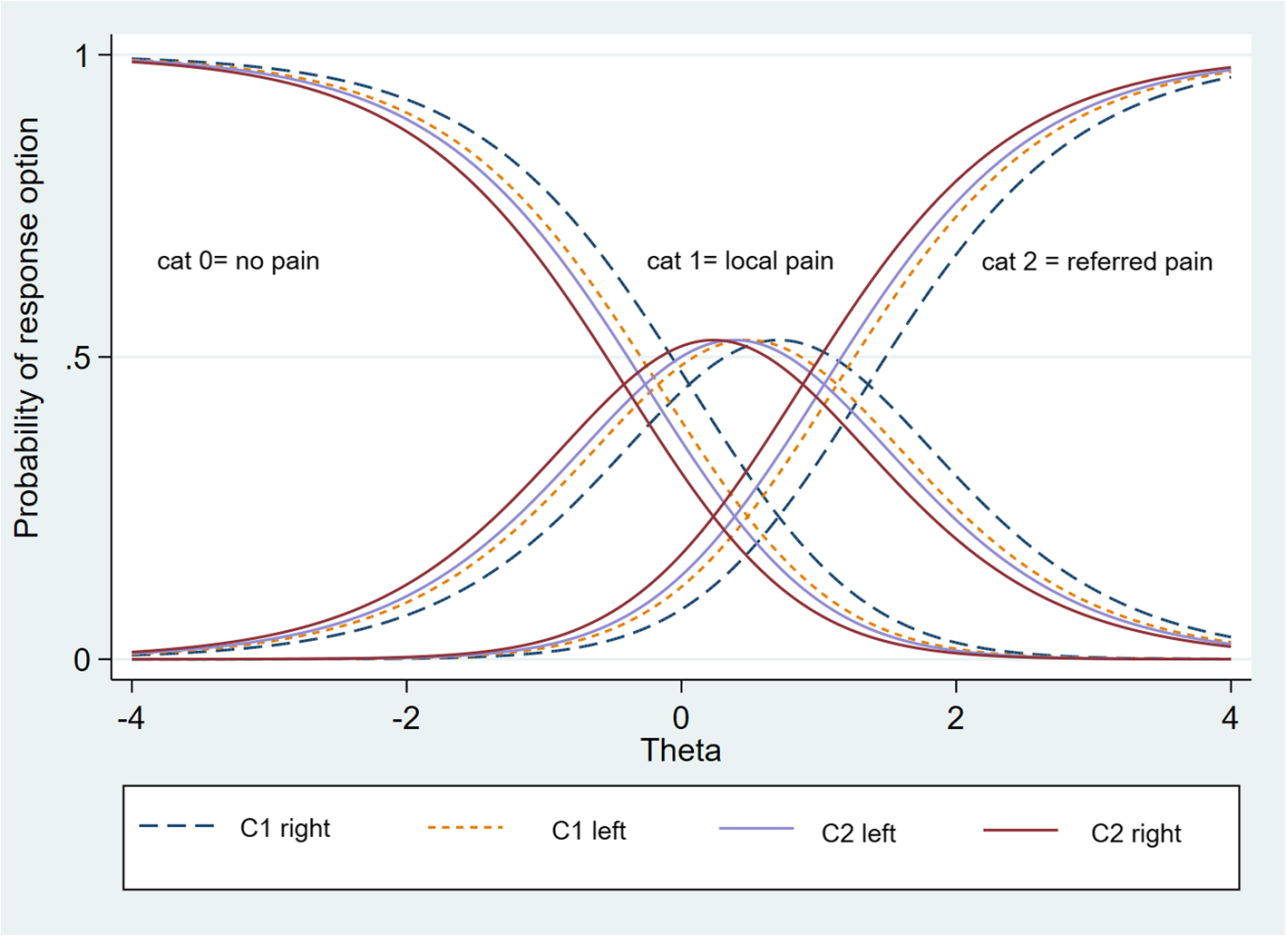
Category characteristic curve displaying the relationship between the probability for one response option (y-axis) and the degree of dysfunction (x-axis). The degree of dysfunction is given in Theta values (mean 0, SD 1). A Theta value of + 4 stands for the highest degree of dysfunction whereas a Theta value of -4 for the lowest. cat = category

Figure 3 displays the category characteristic curve of all four items showing a clear order of the 3 categories (no pain, local pain, referred pain). Only the first option (no pain) and the last option (referred pain) are monotonically decreasing and increasing, respectively. Participants with a low dysfunction (left side of the x-axis) have the highest probability to respond with no pain on palpation of all four palpation points.

In summary the results show that the palpation point C3 can be excluded for subgrouping patients as it does not form one latent construct with the palpation points C1 and C2 and therefore the segments occiput-C2. All palpation points on C1 and C2 are important but C2 has a higher probability to show a painful response in a stratification assessment.

## Discussion

### Stratification

The purpose of this secondary analysis was to identify whether C3 should be added to the palpation procedure when assessing patients with migraine and thereby to confirm the latent construct for stratification. Results showed that for the stratification of migraine patients, as suggested by Luedtke and May [9], the palpation of vertebrae C1 and C2 is sufficient. Palpation of C3, on the other hand, is not clinically represented in one underlying construct. For future studies subgrouping migraine patients according to palpation results, unilateral palpation of C1 and C2 is recommended and C3 can be omitted. It must be considered that the applied ventral pressure can easily have an influence on the adjoining segment and thus also on C2/C3. This is the reason why the exclusion of palpation point C3 for stratification is not equivalent to the exclusion of C3 from the examination of migraine patients. Furthermore, a clear hierarchy between C1 and C2 regarding item difficulty was shown, indicating that patients of this sample were more likely to respond with local or referred pain to palpation of C2 rather than of C1. The groups show a clear order regarding dysfunction, with patients with no pain having the lowest or no dysfunction and patients with referred pain having the highest degree of dysfunction.

Stratification of migraineurs by manual examination has been described in only one study suggesting an associated cervical component in migraine patients differing across subgroups [9]. The rationale behind stratification of migraine patients is to determine whether a clinical subgroup of migraine patients exists in which the cervical spine might have a higher impact on migraine symptoms. Distinguishing these subgroups could guide physicians and therapists to decide efficiently which patient potentially requires treatment of the cervical musculoskeletal system. Knowing which segments exactly can be combined for stratification makes the stratification more robust. However, it must be kept in mind that pain provocation by palpation does not necessarily represent dysfunction of the cervical spine, but could also be a sign of heightened sensitivity i.e. a centrally sensitized TCC [23]. This hypothesis is strengthened by the fact that patients with tension headache can also respond with referred pain to the head [10]. Patients with severe central sensitization and thus allodynia are likely to show tenderness during an attack [24]. Nevertheless, it has been shown, that migraine patients with ictal neck pain show more tenderness of pericranial neck muscles interictally than migraineurs without ictal neck pain, suggesting involvement of peripheral tissue in the pathophysiology [25]. This group showed the same amount of allodynia as the healthy controls. Therefore, and based on the results of this study it could be argued that patients should only be stratified into the local pain group if they respond with local pain at a minimum of two palpation points to exclude patients who only show some soreness due to local sensitization. Equally, it would be more conservative to stratify only patients who respond with referred pain to the head at a minimum of two palpation points to the referred pain group. This approach would reduce the influence of local sensitization. Future studies could use this more conservative stratification to investigate whether migraine patients with referred pain respond differently to interventions and thus constitute a clinically relevant group.

Nevertheless, work by Carvalho and colleagues showed that subgroups differed with respect to the frequency of migraine attacks [26]. Therefore, it can be concluded that the stratification according to Luedtke and May [9] describes clinically relevant subgroups with the group with referred pain to the head being the one with the highest degree of upper cervical dysfunction. The pathophysiological differences of these groups and the influence of the cervical system on migraine symptoms needs to be investigated in further research. The order of the three group categories no pain, local pain and referred pain to the head and the underlying construct was shown to be stable. Therefore, clinical practitioners can stratify patients according to the pain response of each of the four palpation points. The stratification is robust and easier to use in clinical practice than e.g. the calculation of a sum score or theta.

### Stronger connection with the first branch of the trigeminal nerve

A strong association of referred pain is frequently reported between V1 (first branch of the trigeminal nerve) and GON. However, referred or radiating pain is reported much less frequently between V1 and the two branches of the trigeminal nerve V2 and V3, although these are nerve branches of the same nerve, but apparently show less convergence to V1 in the TCC than the GON. Clinically, this difference is reflected in the fact that while up to 76% of migraine patients report neck pain, only 2.3% [27] to 8.9% [28] report facial pain. Thus, it appears that the first branch of the trigeminal nerve is distinct from the rest of the trigeminal system. Furthermore, this makes the first two segments more relevant for migraine patients than C3 as the GON is essentially formed by nerve fibers from the second spinal nerve (C2) and less from C3 [29]. This might be a reason why patients with severe migraine show a higher probability to respond with either local or referred pain to palpation of C2 than of C1 as it has been shown in the current sample.

As early as 1944, it was investigated in healthy subjects how far pain caused by noxious stimuli to the periosteum and muscles from different cervical segments radiates. It was shown that the farther cranially the stimulus was administered, the farther the radiation extended. Stimuli at the level of C2 and C1 reached frontal and orbital regions [11, 30]. These stronger associations of V1 and the upper two cervical segments are supported by the principal component analysis of the present study. The first two segments form a common construct that is reflected in the clinical response.

The present study did not distinguish between migraine patients and healthy subjects. Therefore, results are only valid for the stratification of (severely affected) migraine patients. The examinations of C3, however, might still be important to be included in the manual examination of the cervical region of migraine patients as it might give additional information.

## Conclusion

The results of the principal component analysis show that the manual examination of the cervical spine on migraine patients should be focused on the unilateral palpation of C1 and C2. It was confirmed that these palpation points form a robust latent construct for the stratification of migraine patients. The categories no pain, local pain and referred pain to the head can easily be use clinically to distinguish between migraine patients with low, moderate or high cervical dysfunction. With a future uniform procedure for stratification, the subgroups can be further examined regarding pathophysiological and clinical differences.

## Data Availability

The datasets used and/or analyzed during the current study are available from the corresponding author on reasonable request.

## Abbreviations

AIC: Akaike information criteria
BCC: Boundary characteristic curves
BIC: Bayesian information criterion
BMI: Body Mass Index
C1, C2, C3: Cervical segments 1–3
CM: Chronic migraine
EM: Episodic migraine
Cx: Cervical spine
GON: Greater occipital nerve
HIS: International Headache Society
IRT: Item response theory
LRT: Likelihood ratio test
RSM: Rating scale model
SD: Standard deviation
TCC: Trigeminocervical complex
V1: First branch of trigeminal nerve
